# Intestinal Worms in Children and Their Treatment

**Published:** 1908-03-14

**Authors:** Robert Hutchison


### Hospital Clinics.

### INTESTINAL WORMS IN CHILDREN AND THEIR TREATMENT.

By ROBERT HUTCHISON, M.D., F.R.C.P.

(Abstract of a lecture delivered at the Hospital for Sick Children, Great Ormond Street, on March 5, 1908.)

There are three varieties of worms which may inhabit the alimentary canal of children. These are the tape worm, the round worm, and the thread worm. On rare occasions other forms are met with, but for practical purposes these are the only worms we need discuss as affecting children in this country.

### Tape Worms.

Two varieties of tape worm are found in children. These are the Tænia cucumerina and the Tænia mediocanellata. The first of these is much rarer than the second, and is, in fact, not very well known. It is a comparatively short tape worm, and its presence is recognised by the passage of small oval segments. Both the cases I have hitherto met with were in infants at the breast, as is usual with this worm; this extraordinary point in the distribution of Tænia cucumerina naturally causes difficulty in diagnosis. You may ask, How in the world does a child nursed at the breast get a tape worm? It appears that this particular worm is always acquired from cats, and in every case it is found that the sufferer has been in close association with one of those animals.

Tænia mediocanellata is the common tape worm with which you are all familiar, both in children and in adults. It seldom occurs in young children, but is frequent at and after the second dentition.

Children are often brought by their mothers for advice with regard to worms. Various symptoms are supposed to be caused by their presence, such as wasting in spite of good appetite, abdominal pain, and so on. Let me say at once that, except possibly in the case of thread worms, I know of no symptom that points definitely to the presence of worms. The only method by which you can diagnose the existence of worms in the alimentary canal is by actually seeing segments or entire individuals in the fæces.

### Treatment of Tape Worms.

This is not such an easy matter as one might suppose. You will often fail to get away the whole worm, and this is in many cases because the condition is not taken seriously enough and treatment is half-hearted.

The patient should remain at least twenty-four hours in bed and for some hours before the anthelmintic is given, and until the worm is passed no solid food at all must be allowed. Begin in the evening by giving a purge so as to clear the alimentary canal as much as possible, and thus expose the worm to the direct action of drugs. A full dose of castor oil, or of liquorice powder, or of calomel should be administered: it does not very much matter which, provided the dose is a sufficient one.

Of the anthelmintics none surpasses male fern, which, on account of its nauseous taste, is best given in capsule in 15 grain doses every quarter of an hour for four doses. A child of the age at which tape worm is common will stand such a dose quite well: children, in fact, bear all aperients well in comparison with adults. Having given four such capsules it is best to wait for an hour or so, and then to give another purge to clear out the worm. As a quickly-acting aperient is wanted, an effervescing saline, followed by a hot drink, is useful.

It is very important to attend to details in the passage of the resulting motion. It should be passed into warm water to diminish the risk of breaking the worm. There should be a sheet of black crêpe on the bottom of the vessel, which assists the search for the head of the worm; this can often, with a little practice, be recognised by the naked eye. It is only when the worm is completely evacuated and the head seen that one can be sure of success. Sometimes only part of the worm is passed, and the administration of a dose of morphia to the worm has been suggested, with a view to paralysing the remainder and causing its passage.

If the treatment fails it is not advisable to repeat it until after an interval to allow of growth. The essentials are rest, starvation, purgation, full doses of an anthelmintic, and care in the way in which the worm is received when passed.

About the treatment of Tænia cucumerina I cannot tell you much. Personally I should be rather afraid of giving male fern to a little baby; I should prefer calomel, and perhaps small doses of santonin as well. Both the cases to which I have referred had calomel only, and soon ceased to pass segments, though I had not the opportunity of searching for the head.

### Round Worms.

As in the case of the tape worms, there are no symptoms characteristic of this worm, the Ascaris lumbricoides. There is, however, no doubt that it may cause reflex nervous disturbances of various kinds. I have myself seen cases of convulsions which ceased after the passage of a round worm. These worms are probably derived from certain kinds of food, particularly raw vegetables, such as salads. I once had to do with a family of strict vegetarians in which the father, mother, and both children were suffering from round worms, and I do not doubt that it was from their vegetables that they had contracted the parasites. With a view to prevention it is well to see that anyone who has had a round worm should avoid such foods, or at least take the most careful precautions about washing and cleaning them.

The round worm is the only kind of common worm to which any danger attaches. The danger arises from this reason, that they are always prone to wander. When the worm wanders upwards into the stomach, it is generally promptly vomited; but it may obtain entrance into the larynx and cause suffocation, or into a bronchus and set up bronchiectasis. If a child passes a round worm, it should be at once put through a course of treatment, for these animals often hunt in couples. Fortunately round worms are more easily got rid of than tape worms.

Santonin is the best remedy, for it is effective and safe; amongst numerous other anthelmintics, thymol has been a good deal used. Santonin should be exhibited as a powder in this combination:

| | |
|---|---|
| Santonin ... ... ... ... ... | gr. 1/6 |
| Calomel ... ... ... ... ... | gr. 1/6 |
| Sugar ... ... ... .. ... | q.s. |

Give one such dose for every year of the child's age every night for three or four nights. So much then for round worms: their treatment is simple, and when once the worm is seen there is no anxiety as to any of it having been left behind.

### Thread Worms.

The third and commonest variety of worm that attacks children is the Oxyuris vermicularis. It is very important to realise that thread worms are not a disease in themselves, but a symptom of an unhealthy state of the large bowel. I doubt very much whether a thread worm can exist in a bowel that is healthy. The sort of condition required is one of catarrh associated with the presence of mucus. It is in the folds of the swollen mucous membrane that the worms lodge, and to be successful in treatment this condition must be remedied.

Another difficulty is that the patient, if a child, is constantly re-infecting himself. Further, the ova are often deposited in the vermiform appendix, which forms a regular breeding place for oxyuris. When once one has seen such an appendix full of young worms, it can be realised how difficult it is to stop the constant reinfection.

This brings us to the symptoms caused by thread worms. There are certain purely local effects, such as irritation, scratching, and so on. Beyond these, it is quite possible that incontinence is sometimes due to worms, but there is much less ground for the belief common amongst mothers that picking at the nose is evidence of them. As regards pallor, abdominal pain, wasting, headache, and other vague symptoms, I am convinced that these are really due to the state of ill-health which permits the worms to flourish. It is the children who exhibit Dr. Eustace Smith's "mucous disease," or dyspepsia of the second dentition, as it is also called, that are especially liable to thread worms. The malady is characterised by several of the symptoms just named, and above all by constipation.

To get rid of the worms it is necessary to deal with this underlying condition. One can do this partly by attention to diet, partly by medicines. It is very important to reduce the starchy constituents of the food, such as raw fruit and vegetables, bread, sweets, puddings, and sometimes even potatoes. You must also make sure of a sufficient daily action of the bowels, for which there is nothing better than

| | |
|---|---|
| Pulv. Rhei ... ... ... ... | gr. v. |
| Sod. Bicarb. ... ... ... ... ... | gr. v. |
| Hydrarg. c. Creta ... ... ... | gr. j. |

which is peculiarly efficacious in this condition of the alimentary canal. The value of this powder is probably due to the rhubarb, which has not only an aperient, but also an astringent and tonic afteraction on the mucous membrane. The grey powder has also a share in the result, for when it is omitted the combination is certainly less successful.

The powder should be given regularly for a considerable period, the aim being to produce one loose motion every morning. You may also with advantage give alkalies and bitters before meals. Iron is sometimes of benefit, but must never be given without an aperient.

Some authorities recommend santonin by the mouth for thread worms. I am not at all sure that it has any anthelmintic effect on them, and if you adopt the methods I have indicated there will be no need to use it.

To deal with the worms locally, various injections have been proposed. Infusion of quassia is an old-fashioned prescription, and a very good one; solution of common salt, in the proportion of an ounce to the pint, is another; and infusion of garlic is sometimes very effective. These may be safely used every night for a time. The following may be injected every other night for three doses with good results:

| | |
|---|---|
| Oil of Turpentine ... ... ... ... | ʒij. |
| Santonin ... ... ... ... ... | gr. ij. |
| Starch Mucilage ... ... ... ... | ℥vj. |

There are two different systems of giving an injection for these worms. Some physicians advise a large injection so as to reach as far up the bowel as possible. Others prefer a small one that it may be retained as long as possible. My own feeling is in favour of small injections, which have the advantage that they cause much less discomfort, as the process is one which is frequently repeated; this is an important point when dealing with young children.

The injection should be given warm, and quite slowly, through a funnel. The pelvis of the patient should be raised. An injection of six ounces when given thus may in a child reach the transverse colon by reversed peristalsis. This small quantity is safer and less likely to be painful than the larger doses; in addition, it is far less likely to damage the intestine, whose wall is thinner and more easily ruptured than is often thought.

It is well to order a little weak white precipitate or nitrate of mercury ointment for local application, to relieve itching and to prevent wandering of the worms.

I will only repeat that local measures are less important than attention to the state of the intestine, for if the worms are only killed off locally they will certainly come back, a process which has been known to continue for years.

If I have mentioned to you but a few lines of treatment this afternoon, it is because I believe that if they are thoroughly carried out, satisfactory results will always be obtained.

